# Patterns of Enteral Feeding, Feeding Intolerance, and Mortality in Traumatic Brain Injury: An Observational Study

**DOI:** 10.3390/clinpract16050083

**Published:** 2026-04-26

**Authors:** Hasan M. Al-Dorzi, Abdulaziz R. Al-Qwizani, Turki F. Al-Saikhan, Yousef Alshahwan, Bandar F. Bindayel, Raed Alharthi, Raymond Khan

**Affiliations:** 1 College of Medicine, King Saud Bin Abdulaziz University for Health Sciences, Riyadh 14611, Saudi Arabia; qwizany@outlook.com (A.R.A.-Q.); tfsaikhan@gmail.com (T.F.A.-S.); 421110112@ksau-hs.edu.sa (R.A.); khanra1@mngha.med.sa (R.K.); 2 King Abdullah International Medical Research Center, Riyadh 11481, Saudi Arabia; 3 Intensive Care Department, King Abdulaziz Medical City, Ministry of National Guard Health Affairs, Riyadh 11426, Saudi Arabia

**Keywords:** critical illness, tube feeding, trauma, traumatic brain injury, feeding intolerance, vomiting, gastric residual volume

## Abstract

**Background:** Patients with traumatic brain injury (TBI) are often underfed and frequently experience enteral feeding (EF) intolerance. We examined the association between EF timing, caloric intake and EF intolerance, and mortality. **Methods:** We retrospectively evaluated adult patients with moderate-to-severe TBI in a tertiary-care ICU. In the first 7 days, we recorded daily caloric intake from EF and the occurrence of EF intolerance—defined as a gastric residual volume > 500 mL or >250 mL with vomiting. **Results:** Among 298 patients, 210 (70.4%) received early EF. The median 7-day cumulative caloric intake was 7766 kcal for the early EF group (64.7% of caloric requirement) and 2783 kcal (23.1% of caloric requirement) for the late (after 48 h) EF group (*p* < 0.001). EF intolerance occurred in only 24 patients (8.1%), with no significant difference between the early and late groups. Hospital mortality was 13.8% with early EF versus 30.7% with late EF (*p* = 0.001), 8.5% with caloric intake ≥ 80% of requirement versus 21.3% with lower caloric intake (*p* = 0.02) and 50% in patients with EF intolerance versus 16.1% in those without intolerance (*p* < 0.001). In multivariable logistic regression analysis, early EF was associated with lower mortality (odds ratio 0.326; 95% confidence interval 0.165–0.644), whereas EF intolerance was associated with higher mortality (odds ratio 7.451; 95% confidence interval 2.787–19.922). **Conclusions:** In patients with moderate-to-severe TBI, early EF was associated with higher caloric intake and lower mortality compared to late EF. EF intolerance was uncommon but strongly associated with higher mortality.

## 1. Introduction

Traumatic brain injury (TBI) is associated with hypermetabolic and hypercatabolic states [1], making adequate nutritional support an integral part of patient care [2]. Because of impaired consciousness and swallowing difficulties, critically ill patients with TBI often depend on enteral feeding (EF) during their early stay in the intensive care unit (ICU) to meet their nutritional needs. The timing of initiating EF is important. Delayed EF has been associated with increased infectious complications, and higher mortality rates in critically ill patients [3]. Hence, early EF, initiated within 24–48 h of ICU admission, has been recommended in critically ill patients including those with major trauma, as it may prevent malnutrition, preserve muscle mass, and reduce mortality [3,4,5]. In patients with TBI, a meta-analysis of five randomized controlled trials and three non-randomized prospective studies up to October 2012 found that early feeding, enteral or parenteral, was associated with reduced infection and mortality rates [6]. More recent evidence, however, is mixed. A recent multicenter study of 3080 patients with severe TBI found lower nosocomial pneumonia but similar in-hospital mortality in the early versus late EF groups after propensity score matching [7].

Early and adequate EF is frequently impeded by EF intolerance, which may manifest as vomiting, diarrhea, abdominal distension, constipation, and increased gastric residual volume (GRV) [8]. EF intolerance may affect EF initiation, limit the ability to reach nutritional targets and contribute to nutritional deficiencies [8]. These deficiencies may lead to malnutrition and have been associated with adverse clinical outcomes, including prolonged ICU stay, increased complications, and higher mortality in critically ill patients [8]. Patients with severe TBI may be particularly vulnerable because brain injury triggers a cascade of inflammatory and noninflammatory responses that affect the gastrointestinal tract [2,9,10,11]. This can lead to mucosal alterations, increased permeability and abnormal motility [2,9,10,11]. A study in 882 trauma patients found that patients with TBI (n = 432) had a higher incidence of EF intolerance than those without TBI (18.6% versus 10.4%, *p* < 0.001) [12].

Studies examining the timing of EF and EF intolerance in patients with TBI have yielded conflicting results [2,7,13,14]. Moreover, real-world evidence evaluating the association between EF timing, actual caloric delivery, and EF intolerance with clinically meaningful outcomes in patients with moderate-to-severe TBI remains limited. Therefore, the objectives of this study were to describe practices of EF in patients with moderate-to-severe TBI, determine the rate of EF intolerance and evaluate the association of early versus late EF, caloric intake and EF intolerance with clinical outcomes. We hypothesized that early EF and higher caloric delivery would be associated with lower mortality, whereas EF intolerance would be associated with worse outcomes.

## 2. Materials and Methods

### 2.1. Setting and Patients

This retrospective cohort study evaluated all consecutive adult (aged ≥ 18 years) patients with moderate-to-severe TBI who presented to the emergency department after trauma and were subsequently admitted to the Intensive Care Department of King Abdulaziz Medical City in Riyadh between 1 January 2016 and 31 December 2019. The department had several ICUs which admitted medical, surgical and trauma patients. The units operated as closed ICUs and provided multidisciplinary care in the presence of critical care consultants on a 24/7 basis [15]. Moderate-to-severe TBI was defined as closed or penetrating head trauma leading to Glasgow Coma Scale (GCS) < 13. Moderate TBI was defined as a GCS score of 9–12, whereas severe TBI was defined as a GCS score of 3–8 [16,17]. Patients were excluded if their ICU stay was <3 days, including those who died within the first 3 days, or if brain death was diagnosed within 7 days of ICU admission. All eligible patients were included in the study.

In our ICU, patients with severe TBI were treated with a departmental Head Injury Protocol [18], derived from the guidelines published by the Brain Trauma Foundation [19]. The treating ICU team determined the timing of EF initiation, including the initial hourly volume, taking into consideration associated abdominal injuries, required surgical procedures and clinical stability. The ICU dietician selected the EF formula and determined the caloric goal and hourly EN volume. The ICU nurses used an evidence-based EF protocol [4,20], routinely checked GRV every 4 h by aspirating the feeding tube with a syringe, and documented vomiting episodes, bowel movements and stool consistency. The EF protocol specifies that if GRV is <500 mL and there are no other signs of intolerance, return the aspirate and continue EF at the previous rate, and if GRV is >500 mL or 200–500 mL with other signs of intolerance, return up to 500 mL, hold feeding for 2 h, then resume feeding at the previous rate. The Institutional Review Board of the Ministry of National Guard Health Affairs reviewed and approved this study (approval number: IRB/1720/23), with a waiver of informed consent due to its nature.

### 2.2. Data Collection

We collected data on demographic information, GCS score on presentation, Injury Severity Score (ISS) [21], findings of the initial brain computed tomography, non-TBI injuries, use of the departmental Head Injury Protocol and intracranial pressure monitoring, and pertinent laboratory results on admission (white blood cell count, hemoglobin, platelet count, serum creatinine, serum lactate and cultured respiratory pathogens). We recorded the following nutritional data from day 1 through day 7: use of EF and parenteral nutrition, EF formula, GRV on every check, episodes of vomiting and witnessed aspiration, the number of bowel movements, and the use of prokinetics (metoclopramide and erythromycin). This 7-day period was selected as it overlaps the early phase of critical illness and the hypermetabolic response in moderate-to-severe TBI, when invasive support, such as invasive mechanical ventilation and intracranial pressure management, is typically required [22,23], and EF is probably most relevant.

In this study, we defined early EF as EF initiation within 48 h of ICU admission and delayed EF as initiation after 48 h. We also evaluated different manifestations of EF intolerance, including high GRV, vomiting and diarrhea [8]. The main definition of EF intolerance was a GRV > 500 mL or 250 mL accompanied with vomiting [8]. We did not include vomiting with GRV < 250 mL in the EF intolerance definition. We defined diarrhea as having three or more loose bowel movements per day; however, it was not included in the EF intolerance definition, due to its multifactorial etiology, the absence of a uniform definition, and the lack of quantitative assessment (amount/weight) of stool in the medical records [8].

We classified EF formulas as specialized, non-specialized and energy-dense (≥1.5 kcal/mL). Based on the volume of EF and the formula used, we calculated the provided energy on days 1 to 7. Caloric requirements were calculated using the American College of Chest Physicians predictive equation (25 kcal/kg actual body weight for the nonobese and 14 kcal/kg actual body weight for the obese [body mass index ≥ 30 kg/m^2^]) [4]. We also calculated the cumulative caloric intake from EF over the first 7 ICU days and the corresponding percentage of the calculated caloric requirement as well as the protein intake from the EF formulas.

In this study, the primary outcome was hospital mortality. The secondary outcomes were ICU mortality, GCS at discharge for hospital survivors, ICU-acquired pneumonia, duration of mechanical ventilation, and length of stay in the ICU and hospital.

### 2.3. Statistical Analysis

We divided patients into early versus late EF groups and EF intolerance versus no EF intolerance groups. We summarized categorical variables as frequencies with percentages, and continuous variables as medians with interquartile ranges (IQR). The normality of distribution of continuous variables was assessed using skewness and kurtosis statistics. We did not impute missing data. Chi-square or Fisher’s exact test was used to compare between-group differences in categorical variables. Student *t*-test or Mann–Whitney U test was employed to compare between-group differences in continuous variables, as appropriate. We assessed the patients who received ≥80% of energy requirements during the first 7 ICU days. Although arbitrary, this threshold reflects energy target intake for ICU patients.

We performed multivariable logistic regression analysis to assess the independent association of early versus late EF and manifestations of EF intolerance with hospital mortality, while adjusting for potential confounders. The independent variables entered in the model were clinically relevant and were age, admission GCS, ISS, timing of EF initiation (early versus late), and the presence of EF intolerance, vomiting or diarrhea in the first 7 ICU days. None of the variables entered in the models had missing data. We checked for collinearity between the continuous variables and found it to be nonsignificant (variance inflation factor of 1.0). We performed sensitivity analysis where we entered early EF initiated within 24 h instead of the primary definition of 48 h as an independent variable. In addition, the model was re-run in subgroups of patients who received Head Injury Protocol and those without abdominal injury. We presented the results as odds ratio (OR) with 95% confidence interval (CI). We also performed receiver operating characteristic (ROC) curve analysis to evaluate the relationship between cumulative caloric intake and hospital survival. The Youden index was used to assess the caloric intake that best discriminated between survivors and non-survivors [24].

We adjusted for multiple comparisons of the secondary outcomes using the false discovery rate method to reduce the risk of type I error. All statistical tests were considered significant if the *p*-value was <0.05. SPSS software version 15 (IBM Corp., Armonk, NY, USA) was used for all statistical analyses.

## 3. Results

### 3.1. Characteristics of Patients

During the 4-year study period, 298 patients were admitted to the ICU with moderate-to-severe TBI. Their median age was 28.0 years (IQR 23.0, 35.0), and most patients (95.6%) were males. On admission, the median GCS score was 6 (IQR 3, 8) with 241 patients (80.9%) having severe TBI. The median ISS was 30 (IQR 25, 36). Concomitant abdominal injuries were present in 31 patients (10.4%). All patients required mechanical ventilation on admission. Most patients (245/298, 82.2%) were managed according to the Head Injury Protocol, and 61 patients (20.5%) underwent intracranial pressure monitoring.

### 3.2. Feeding Data

Most patients (n = 210, 70.4%) received early EF within 48 h of injury (104 patients [34.9%] within 24 h). Compared with the late EF group (n = 88), the early EF group had similar age and admission GCS but were more likely to have isolated head injury (*p* = 0.04) and less likely to have concomitant abdominal injury (6.7% versus 14.8%, *p* = 0.03) (Table 1). No patient received parenteral nutrition during the study period.

Table 2 summarizes feeding practices in the first 7 days of ICU stay. Specialized EF formulas were used in <20% of patients and energy-dense formulas (specialized and non-specialized) in approximately 30% of patients with no significant difference between the early and late EF groups. Compared with the late EF group, patients in the early EF group received significantly higher caloric intake on all 7 days (*p* < 0.001), resulting in a higher cumulative caloric intake (7766 kcal [IQR 5677, 9227], corresponding to 64.7% [IQR 45.9, 82.4] of caloric requirement, in the early EF group versus 2783 kcal [IQR 164, 5223], corresponding to 23.1% [IQR 1.3, 41.0] of caloric requirement, in the late EF group; *p* < 0.001). Only 59 patients received ≥80% of the calculated caloric requirement during the 7-day study period. The early EF group also received higher cumulative protein intake from the EF formula (316 g [IQR 232, 371], corresponding to 0.6 g/kg/day [IQR 0.4, 0.7], versus 143 g [IQR 71, 232], corresponding to 0.3 g/kg/day [IQR 0.1, 0.5], in the late group; *p* < 0.001).

### 3.3. Enteral Feeding Intolerance

Table 2 also summarizes findings related to EF intolerance. Ninety-four patients (31.5%) had GRV > 250 mL and 20 (6.7%) > 500 mL at least once in the first 7 ICU days. Twenty-three patients (7.7%) had at least one episode of vomiting in the first 7 days (17/210 [8.1%] in the early EF group versus 6/88 [6.8%] in the late EF group, *p* = 0.71). Vomiting episodes were most common on day 3 (4.5%) in the late EF group and on day 7 (3.3%) in the early EF group. Diarrhea was more frequent than vomiting, occurring in 113 out of 298 patients (37.9%). More patients in the early EF group had diarrhea (92/210 [43.8%] versus 21/88 [23.9%] for the late EF group, *p* = 0.001). Diarrhea was most frequent on day 7 in both the early and late EF groups (20.0% and 10.2%, respectively).

Only 24 out of 298 patients (8.1%) met the study definition of EF intolerance (any GRV > 500 mL or GRV > 250 mL accompanied with vomiting in the first 7 days of ICU stay). The daily prevalence of EF intolerance ranged from 0.5 to 1.9% in the early EF group and 0 to 3.4% in the late EF group (Table 2). Patients with EF intolerance had similar demographics and injury characteristics compared with those without intolerance, except for higher white blood cell counts and creatinine levels (Table 1). EF intolerance occurred in 19 of 210 patients (9.0%) in the early EF group and 5 of 88 patients (5.7%) in the late group (*p* = 0.33).

Prokinetic agents were used frequently in patients with EF intolerance. Metoclopramide was provided to 22 out 24 patients (91.7%) and erythromycin was added to metoclopramide in five (20.8%).

### 3.4. Outcomes

The outcomes of patients are shown in Table 3. Fifty-six patients (18.8%) died in the hospital, all in the ICU. The mortality rate was 13.8% for the early EF group and 30.7% for the late EF group. On multivariable logistic regression analysis, early versus late EF (OR, 0.326; 95% CI, 0.165–0.644) and EF intolerance (OR, 7.451; 95% CI, 2.787–19.922) were independently associated with hospital mortality in the whole study cohort (Table 4). Similar findings were observed in patients who received Head Injury Protocol (early versus late EF: OR, 0.216; 95% CI, 0.081–0.572; EF intolerance: OR, 7.169; 95% CI, 2.473–20.787) and in patients without abdominal injury (early versus late EF: OR, 0.197, 95% CI, 0.0.74–0.521; EF intolerance: OR, 8.568; 95% CI, 2.898–25.329). Early EF within 24 h versus later EF was also associated with lower mortality risk (OR, 0.185; 95% CI, 0.071–0.479). Neither vomiting nor diarrhea was associated with mortality.

Among patients who received early EF, the use of energy-dense formulas on day 1 or 2 was associated with similar hospital mortality compared with standard formula (6/65 [9.2%] and 23/145 [15.9%], respectively; *p* = 0.20).

**Figure 1:**
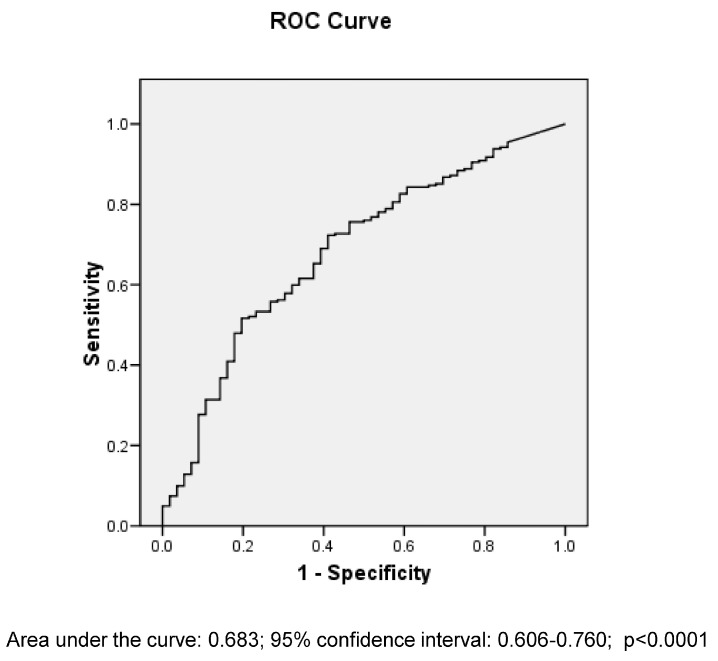
Receiver operating characteristic curve analysis for ability of the cumulative caloric intake (percent of predicted caloric requirement) in the 7-day study period to predict hospital survival.

The 59 patients who received ≥80% of their caloric requirement during the 7-day study period had a mortality rate of 8.5% compared with 21.3% among the 239 patients who received less calories (*p* = 0.02). ROC curve analysis found that caloric intake (percent of calculated caloric requirement) was significantly associated with hospital mortality, with modest discriminative ability (area under the curve 0.68; 95% CI, 0.61–0.76) (Figure 1). The optimal discriminatory threshold based on the highest Youden index (0.31) corresponded to two caloric intake values: 40% and 59% of caloric requirement.

Hospital mortality was also higher in the patients who had EF intolerance (50% versus 16.1% for those who did not have intolerance, *p* < 0.001). For the 94 patients who had any GRV > 250 mL, the hospital mortality was 24.5% compared with 16.2% for the other patients (*p* = 0.09). Hospital mortality did not differ between patients with vomiting and those without (3/23 [13.0%] versus 53/275 [19.3%]; *p* = 0.59) and between patients with diarrhea and those without (20/113 [17.7%] versus 36/185 [19.5%]; *p* = 0.71).

Among the 298 study patients, 49 (16.4%) had ICU-acquired pneumonia with no significant differences between the early and late EF groups (17.6% and 13.6, respectively; *p* = 0.40) and the EF intolerance and no-intolerance groups (8.3% and 17.2%, respectively; *p* = 0.39). The median duration of mechanical ventilation was 10 days (IQR 6, 14). The median length of stay was 13 days (IQR 7, 20) in the ICU and 28 days (IQR 12, 64) in the hospital.

## 4. Discussion

In this study of patients with moderate-to-severe TBI, early EF within 48 h of admission was provided to 70.4% of patients and was associated with higher caloric intake compared with late EF. EF intolerance occurred in 8.1% of patients. Early EF was associated with lower mortality on multivariable logistic regression. Patients with EF intolerance had a markedly high mortality rate (50%).

EF is an essential component of critical care, as malnutrition and energy deficits have been shown to be risk factors for morbidity and mortality in critically ill patients. Early EF within 48 h is recommended for most critically ill patients who cannot take oral diet, including those with TBI [4,5,19]. In our study, we found that EF was started early in more than two thirds of patients with moderate-to-severe TBI. Patients with concomitant abdominal injury were more likely to have delayed initiation of EF. Early EF has several benefits in critically ill patients including the preservation of gut mucosal integrity and maintaining gastrointestinal homeostasis [25]. We found that EF initiation within 24 h or within 48 h compared with later initiation was associated with a lower risk of hospital mortality in the multivariable logistic regression analysis. This association should be interpreted cautiously, given the inherent limitations of observational data and the lack of important clinical variables, such as illness severity. As such, these findings should not be viewed as evidence of a causal relationship. The relationship between EF timing and mortality in patients with TBI is inconsistent across the literature [6,7,13,14,26]. Earlier observational and interventional studies generally suggested a benefit of early EF [6]. More recently, a single-center study of 1353 neurosurgical patients, including those with TBI, reported that EF initiated within 72 h was associated with lower risk of infections and mortality in unadjusted and adjusted analysis [26]. In contrast, a multicenter study of 3080 patients with severe TBI found similar hospital mortality in early versus late EF groups on propensity score-matched analysis [7]. These heterogeneous results highlight ongoing uncertainty regarding the optimal EF timing in TBI. Differences in patient populations, study design, and analytic methods may partly explain the observed variability in outcomes.

Consistent with previous reports, we found that patients with TBI were frequently underfed. In our study, cumulative caloric intake reached 64.7% of caloric requirement with early EF and 23.1% with late EF. Although higher caloric intake was associated with lower hospital mortality in our study, its ability to predict mortality was modest (area under ROC curve 0.68), suggesting limited clinical utility. These findings are partly consistent with results from an international study of 1045 patients from 341 ICUs in which 94% of patients with TBI had EF but received only 58% of estimated caloric requirement [27]. While caloric intake was not associated with mortality in that study, a greater caloric deficit was associated with longer time until discharge alive from both ICU and hospital [27]. Caloric intake and deficit are influenced by multiple factors, including enteral and parenteral nutrition practices, use of EF protocols, EF interruptions, EF intolerance, and use of prokinetics [28,29]. Each of these factors may vary across studies and may contribute to differences in reported caloric intake and outcomes.

EF intolerance is common in critically ill patients, with a systematic review of 72 studies estimating its prevalence at 38% (95% CI, 31–46%) [30]. The reported rates vary widely due to heterogeneity in definitions and assessed manifestations, which commonly include delayed gastric emptying, abdominal distension, vomiting, and diarrhea [8,30]. Although GRV is among the most commonly used indicators of EF intolerance, it primarily reflects gastric emptying and may lack specificity for the dysfunction of the entire gastrointestinal tract [8,30]. In our study, we evaluated different manifestations of EF intolerance and observed a relatively low rate of EF intolerance (8.1%) when defined by the presence of high GRV. A higher rate (18.6%) was seen in a study of 432 patients with TBI [12]. The lower rate in our study may be related to the use of a restrictive definition for EF intolerance and to the generally low EF intake in the majority of patients. Diarrhea was common in our study, but it could be related to reasons other than EF intolerance. We also found that the rate of EF intolerance was similar in patients with early and late EF. This supports initiating EF early in patients with TBI. In critically ill patients, EF intolerance has been associated with adverse outcomes, including nutrition inadequacy, longer ICU stay, and higher mortality [30,31]. In the current study, and in agreement with previous reports, patients with EF intolerance had high mortality (50%), and EF intolerance—but not vomiting or diarrhea—was independently associated with hospital mortality in multivariable logistic regression analysis. This association should not be interpreted as causal, as EF intolerance likely reflects underlying organ dysfunction, physiological deterioration and higher illness severity, which could explain its association with higher mortality [32].

The study results should be interpreted in light of its strengths and limitations. Strengths include the relatively large sample size and the evaluation of different manifestations of EF intolerance. It provides valuable insights into the optimal nutritional management of TBI, a common condition with high morbidity and mortality in Saudi Arabia and the Middle East [33]. However, several limitations should be acknowledged. The retrospective and single-center design limits causal inference and may introduce selection bias and confounding by indication, as decisions on EF initiation and progression were likely influenced by clinical judgment and patient stability rather than random allocation. The generalizability of our findings is also limited by the baseline characteristics of our cohort, including the predominance of young male patients, variability of nutrition practices across ICUs [29], and the use of a narrow definition of EF intolerance. We primarily defined EF intolerance based on GRV, which may not reflect the function of the entire gastrointestinal system, and more importantly, its ability to safely absorb macro- and micro-nutrients [8]. This may affect comparability with other studies that used broader definitions. Including all signs of gastrointestinal dysfunction in the EF intolerance definition may decrease specificity and lead to over-diagnosis. Although the development and validation of reliable biomarkers of EF intolerance could enhance diagnostic accuracy, such tools are currently lacking in clinical practice. The low rate of EF intolerance in our study prevented us from performing multivariable regression analysis to evaluate its predictors. Additionally, associations of EF timing, caloric intake and EF intolerance with mortality may be related to unmeasured confounders. The multivariable regression model used in this study was likely insufficient as important variables—such as baseline nutritional status, hemodynamic instability, and organ failure scores not captured by ISS—were not included, increasing the likelihood of residual confounding. As a result, the observed association between EF timing and mortality may reflect unmeasured differences in illness severity or clinical trajectories rather than a true effect of early EF. We also lacked data on protein intake from sources other than EF formulas, feeding interruptions and feeding tube location (intra-gastric or post-pyloric), all of which are important factors, which may significantly affect both feeding adequacy and outcomes. The Brain Trauma Foundation suggests post-pyloric EF to reduce ventilator-associated pneumonia [19]. Nevertheless, a recent large international prospective study found that gastric feeding was the predominant route (94.1%) in 1691 neurologically injured patients with higher caloric intake in the gastric versus small bowel feeding group and similar hospital mortality [34].

## 5. Conclusions

In patients with moderate-to-severe TBI, early EF within 48 h of admission was associated with higher caloric intake in the first 7 days of ICU stay and lower mortality compared with late EF. Our results are consistent with current guidelines that advocate early initiation of EF within 24–48 h in critically ill patients, including those with TBI, when clinically feasible in the absence of contraindications. EF intolerance, defined by high GRV with or without vomiting, was uncommon (8.1%) in our study and was not more frequent with early EF. However, its presence was associated with adverse outcomes, whereas vomiting and diarrhea were not. While causality cannot be inferred, our results suggest that timely initiation of EF and careful monitoring for EF intolerance may be important components of nutritional management in critically ill patients with TBI. Further prospective studies are warranted to better define optimal feeding strategies and to clarify the mechanisms linking EF intolerance with adverse outcomes.

## Figures and Tables

**Table 1:** Baseline demographic and clinical characteristics of patients with moderate-to-severe traumatic brain injury, stratified by enteral feeding timing (early: initiation within 48 h of admission to the intensive care unit; late: initiation after 48 h) and feeding intolerance.

	All Patients N = 298	Early EF N = 210	Late EF N = 88	*p*-Value	EF Intolerance N = 24	No EF Intolerance N = 274	*p*-Value
Age (years), median (IQR)	28.0 (23.0, 35.0)	28.0 (23.0, 35.0)	29.5 (22.3, 35.0)	0.61	28.5 (24.0, 32.5)	28.0 (23.0, 35.0)	0.89
BMI (kg/m^2^), median (IQR)	24.9 (22.0, 27.8)	25.4 (22.0, 27.9)	23.9 (21.7, 27.8)	0.12	24.7 (22.1, 29.3)	24.9 (22.0, 27.8)	0.99
Male sex, N (%) Female sex, N (%)	285 (95.6) 13 (4.4)	200 (95.2) 10 (4.8)	85 (96.6) 3 (3.4)	0.76	24 (100) 0 (0)	261 (95.3) 13 (4.7)	0.61
Chronic diseases, N (%)							
Hypertension	6 (2.0)	1 (0.5)	5 (5.7)	0.01	0 (0)	6 (2.2)	1.0
Diabetes	3 (1.0)	1 (0.5)	2 (2.3)	0.02	0 (0)	3 (1.1)	1.0
ISS, median (IQR)	30 (25, 36)	27 (25, 34)	34 (27, 43)	<0.001	27 (25, 34)	30 (25, 36)	0.30
Active bleeding on admission, N (%)	170 (57.0)	110 (52.4)	60 (68.2)	0.01	15 (62.5)	155 (56.6)	0.57
Details of brain injury							
Admission GCS, median (IQR) Admission GCS < 9 (severe TBI), N (%)	6 (3, 8) 241 (80.9)	6 (3, 8) 166 (79.0)	6 (3, 8) 75 (85.2)	0.36 0.22	5 (3, 7) 22 (91.7)	6 (3, 8) 219 (79.9)	0.20 0.16
Intracerebral hemorrhage, N (%)	211 (70.8)	152 (72.4)	59 (67.0)	0.36	19 (79.2)	192 (70.1)	0.35
Cerebral contusion, N (%)	113 (37.9)	87 (41.4)	26 (29.5)	0.054	7 (29.2)	106 (38.7)	0.36
Epidural hematoma, N (%)	31 (10.4)	23 (11.0)	8 (9.1)	0.63	2 (8.3)	29 (10.6)	1.0
Subdural hematoma, N (%)	118 (39.6)	82 (39.0)	36 (40.9)	0.76	13 (54.2)	105 (38.3)	0.13
Subarachnoid hemorrhage, N (%)	117 (39.3)	85 (40.5)	32 (36.4	0.51	10 (39.3)	107 (39.10	0.80
Isolated head injury, N (%)	71 (23.8)	57 (27.1)	14 (15.9)	0.04	7 (29.2)	64 (23.4)	0.52
Concomitant abdominal injury, N (%)	31 (10.4)	17 (8.1)	14 (15.9)	0.04	3 (12.5)	28 (10.2)	0.73
Laboratory findings on admission							
White blood cell count (10^6^/L), median (IQR)	16.2 (12.1, 20.3)	16.0 (12.4, 20.0)	16.5 (11.5, 20.6)	0.52	17.9 (14.8, 23.7)	16.0 (11.8, 19.8)	0.01
Platelet count (10^9^/L), median (IQR)	254 (199, 320)	257 (204, 326)	246 (179, 293)	0.02	281 (207, 331)	252 (197, 314)	0.43
Hemoglobin (g/L), median (IQR)	141 (122, 155)	142 (123, 155)	136 (118, 153)	0.06	143 (128, 156)	140 (122, 155)	0.18
Lactate (mmol/L), median (IQR)	2.0 (1.1. 3.4)	1.8 (1.1, 3.1)	2.5 (1.1, 4.1)	0.06	2.5 (1.3, 4.7)	2.0 (1.1, 3.3)	0.17
INR, median (IQR)	1.1 (1.1, 1.3)	1.1 (1.1, 1.2)	1.2 (1.1, 1.3)	0.03	1.1 (1.1, 1.3)	1.1 (1.0, 1.3)	0.53
Creatinine (*μ*mol/L), median (IQR)	81 (69, 96)	79 (68, 94)	85 (74, 99)	0.02	93 (78, 103)	80 (68, 94)	0.046
Management in the first 24 h							
Head injury protocol, N (%)	245 (82.2)	178 (84.8)	67 (76.1)	0.08	22 (91.7)	223 (81.4)	0.27
ICP monitoring with/without craniotomy, N (%)	61 (20.5)	48 (22.9)	13 (14.8)	0.12	6 (25.0)	55 (20.1)	0.60

BMI: body mass index; EF: enteral feeding; GCS: Glasgow Coma Scale; ICP: intracranial pressure; INR: International Normalized Ratio; IQR: interquartile range; ISS: Injury Severity Score; TBI: traumatic brain injury. Missing data: BMI (n = 1), white blood cell count (n = 1), platelet count (n = 1), hemoglobin (n = 1), lactate (n = 50), INR (n = 6), creatinine (n = 2).

**Table 2:** Data on feeding practices, caloric intake and enteral feeding intolerance during the first 7 days of intensive care unit stay.

Formula	Day 1		Day 2		Day 3		Day 4		Day 5		Day 6		Day 7	
	Early EF	Late EF	Early EF	Late EF	Early EF	Late EF	Early EF	Late EF	Early EF	Late EF	Early EF	Late EF	Early EF	Late EF
No feeding, N (%)	106 (50.5)	88 (100)	1 (0.5)	88 (100)	7 (3.3)	50 (56.8)	13 (6.2)	47 (53.4)	19 (9.0)	30 (34.1)	27 (12.9)	27 (30.7)	34 (16.2)	30 (34.1)
EF, N (%)	104 (49.5)	0 (0)	209 (99.5)	0 (0)	200 (95.2)	34 (38.6)	195 (92.9)	41 (46.6)	188 (89.5)	55 (62.5)	178 (84.8)	58 (65.9)	169 (32.9)	54 (61.4)
Oral diet, N (%)	0 (0)	0 (0)	0 (0)	0 (0)	3 (1.4)	4 (4.5)	2 (0.9)	0 (0)	3 (1.4)	3 (3.4)	5 (2.4)	3 (3.4)	7 (3.3)	4 (4.5)
Feeding formula, N (%) Regular Specialized	89 (85.6) 15 (14.4)	0 (0) 0 (0)	174 (83.3) 35(16.7)	0 (0) 0 (0)	164 (82.0 36 (18.0)	30 (88.2) 4 (11.8)	159 (81.5) 36 (18.5)	37 (90.2) 4 (9.8)	154 (81.9) 34 (18.1)	49 (89.1) 6 (10.9)	147 (82.6) 31 (17.4)	52 (89.7) 6 (10.3)	139 (82.2) 30 (17.8)	47 (87.0) 7 (13.0)
Energy-dense formula^c^, N (%)	34 (32.7)	0 (0)	64 (30.6)	0 (0)	59 (29.5)	10 (29.4)	59 (30.3)	15 (36.6)	59 (31.4)	23 (41.8)	58 (32.6)	26 (44.8)	50 (29.6)	23 (42.6)
EF volume/day (mL) ^a^	0 (0, 296)	0 (0, 0)	635 (288, 1119)	0 (0, 0)	1114 (770, 1390)	0 (0, 280)	1140 (720, 1440)	0 (0, 736)	1173 (513, 1572)	334 (0, 1103)	1161 (503, 1560)	550 (0, 1160)	1145 (384, 1577)	405 (0, 1267)
EF calories/day ^a^	0 (0, 318)	0 (0, 0)	720 (338, 1358)	0 (0, 0)	1344 (836, 1620)	0 (0, 337)	1386 (875, 1689)	0 (0, 955)	1443 (713, 1768)	339 (0, 1207)	1440 (671, 1722)	585 (0, 1489)	1435 (520, 1781)	509 (0, 1529)
EF calories/kg/day	0 (0, 4.1)	0 (0, 0)	9.5 (4.8, 17.7)	0 (0, 0)	17.5 (11.3, 22.2)	0 (0, 3.8)	17.9 (11.1, 23.1)	0 (0, 13.1)	18.5 (9.5, 24.9)	5.2 (0, 17.9)	18.3 (8.8, 24.3)	8.0 (0, 20.6)	18.7 (7.1, 23.8)	7.3 (0, 19.9)
Max GRV (mL)/check ^b^	0 (0, 20)	0 (0, 0)	30 (0, 100)	0 (0, 38)	45 (10, 150)	10 (0, 100)	50 (0, 135)	0 (0, 100)	50 (0, 133)	15 (0, 100)	25 (0, 110)	15 (0, 90)	30 (0, 100)	5 (0, 60)
Total GRV (mL)/day	0 (0, 20)	0 (0, 0)	50 (0, 200)	0 (0, 55)	105 (10, 322)	10 (10, 141)	100 90, 300)	0 (0, 200)	103 (0, 300)	20 (0, 240)	55 (0, 333)	23 (0, 150)	60 (0, 276)	10 (0, 140)
Patients with vomiting ^c^ , N (%)	0 (0)	1 (1.1)	2 (1.0)	2 (2.3)	3 (1.4)	4 (4.5)	3 (1.4)	3 (3.4)	3 (1.4)	1 (1.1)	1 (0.5)	0 (0)	7 (3.3)	1 (1.1)
Patients with diarrhea ^d^ , N (%)	2 (1.0)	0 (0)	7 (3.3)	2 (2.3)	16 (7.6)	0 (0)	15 (7.1)	4 (4.5)	21 (10.0)	5 (5.7)	28 (13.3)	8 (9.1)	42 (20.0)	9 (10.2)
Patients with witnessed aspiration ^c^ , N (%)	14 (6.7)	4 (4.5)	8 (3.8)	2 (2.3)	7 (3.3)	2 (2.3)	8 (3.8)	2 (2.3)	7 (3.3)	2 (2.3)	8 (3.8)	2 (2.3)	7 (3.3)	3 (3.4)
Patients with EF intolerance, N (%)	1 (0.5)	0 (0)	3 (1.4)	0 (0)	4 (1.7)	1 (1.1)	3 (1.4)	1 (1.1)	4 (1.9)	0 (0)	3 (1.4)	3 (3.4)	4 (1.9)	3 (3.4)

EF: enteral feeding; GRV: gastric residual volume. Continuous variables are presented as median with interquartile range. ^a^ For EF volume and caloric intake, the early EF group had significantly higher values on all days (*p* < 0.001). ^b^ For GRV per check, the value was significantly higher in the EF group on all days expect day 5 (*p* = 0.07) and day 6 (*p* = 0.18). ^c^ For the use of energy-dense formula, vomiting and witnessed aspiration, there were no significant differences between the early and late EF groups on any day (*p* > 0.05). ^d^ For diarrhea, it was more statistically more common only in the early EF group on day 3 (*p* = 0.004) and day 7 (*p* = 0.04).

**Table 3:** Outcomes of patients with moderate-to-severe traumatic brain injury.

Outcome Variable	All Patients N = 298	Early EF N = 210	Late EF N = 88	*p*-Value (Adjusted *)	EF Intolerance N = 24	No EF Intolerance N = 274	*p*-Value (Adjusted *)
Hospital mortality, N (%)	56 (18.8)	29 (13.8)	27 (30.7)	0.001	12 (50)	44 (16.1)	<0.001
ICU mortality, N (%)	56 (18.8)	29 (13.8)	27 (30.7)	0.001 (0.01)	12 (50)	44 (16.1)	<0.001 (0.002)
ICU-acquired pneumonia, N (%)	49 (16.4)	37 (17.6)	12 (13.6)	0.40 (0.46)	2 (8.3)	47 (17.2)	0.39 (0.49)
Tracheostomy, N (%)	73 (24.5)	53 (25.2)	20 (22.7)	0.65 (0.65)	69 (25.2)	4 (16.7)	0.35 (0.49)
GCS at discharge for survivors, median (IQR)	15 (13, 15)	14 (11, 15)	15 (14, 15)	0.003 (0.01)	14 (9, 15)	15 (13, 15)	0.42 (0.49)
Hospital stay (days), median (IQR)	28 (12, 64)	29 (16, 64)	23 (8, 65)	0.06 (0.13)	15 (9, 62)	29 (13, 65)	0.08 (0.28)
ICU stay (days), median (IQR)	13 (7, 20)	13 (8, 19)	11 (5, 24)	0.18 (0.24)	11 (5, 18)	13 (7, 21)	0.27 (0.49)
Mechanical ventilation (days), median (IQR)	10 (6, 14)	11 (7, 13)	9 (4, 15)	0.15 (0.24)	9 (6, 15)	10 (6, 14)	0.77 (0.77)

Ef: enteral feeding; GCS: Glasgow Coma Scale; ICU: intensive care unit; IQR: interquartile range. * Benjamini–Hochberg adjusted *p*-value with false discovery rate set at 0.05. Adjusted *p*-value is significant if <0.05.

**Table 4:** Multivariable logistic regression analysis the predictors of hospital mortality.

Variable	Odds Ratio	95% Confidence Interval	*p*-Value
Age per 1-year increment	1.011	0.985–1.038	0.41
Glasgow Coma Scale on admission per 1-unit increment	0.757	0.657–0.871	<0.001
Injury Severity Score per 1-unit increment	1.011	0.985–1.039	0.40
Early (within 48 h) versus late enteral feeding	0.326	0.165–0.644	0.001
Enteral feeding intolerance in the first 7 days	7.451	2.787–19.922	<0.001
Any vomiting in the first 7 days	0.599	0.153–2.342	0.46
Any diarrhea in the first 7 days	0.968	0.483–1.941	0.93

The variables entered in the model were age, Injury Severity Score, admission Glasgow Coma Scale, timing of enteral feeding initiation (early versus late), and the presence of enteral feeding intolerance, vomiting or diarrhea in the first 7 ICU days. Enteral feeding intolerance was defined as a gastric residual volume > 500 mL or 250 mL with vomiting. The model was statistically significant compared with the null model (chi-square value = 48.5, *p* < 0.001) and had a correct classification rate of 83.2%. The Hosmer and Lemeshow goodness-of-fit test was acceptable (*p* = 0.30). Nagelkerke R^2^ = 0.242.

## Data Availability

The data presented in this study are available on request from the corresponding author. The data are not publicly available due to institutional policies.
